# Of mentors, apprenticeship, and role models: a lesson to relearn?

**DOI:** 10.3402/meo.v19.25428

**Published:** 2014-08-21

**Authors:** Shahla Siddiqui

**Affiliations:** Khoo Teck Puat Hospital, Singapore

From early in their careers, modern-day physicians are confronted with a myriad of challenges in meeting increasing public expectations of skill, judgment, and expertise (1, 2). At the same time, physicians’ career satisfaction and success – even in academic medical centers – are indelibly linked to outcomes and productivity. Given these competing pressures, are the traditional, ‘tried-and-true’ approaches to medical training adequate or even compatible with this changing context?

Historically, the role of mentors (and mentorship) in medicine has been seen as instrumental in achieving a balanced, successful personal and professional life (3). The explicit and implicit transference of knowledge, craft, and ‘wisdom’ can lessen mentees’ learning curves and provide satisfaction for the mentor. However, in this day and age when administrative, clinical, and research demands weigh heavily on the time of potential mentors, it begs the question: Are professionals truly being allowed to ‘indulge’ in the luxury of mentorship? Moreover, what beneficial aspects of ‘apprenticeship’ are sacrificed? Do current ‘role models’ suffice for mentees seeking inspiration from senior colleagues? (4, 5).

The word ‘mentor’ is derived from Greek mythology. In Homer's *Odyssey*, Mentor was a friend of Ulysses entrusted with the welfare and guidance of his son, Telemachus. Mentor, assisted by the goddess Athena, was empowered in this exalted role to shape this young man into the new king (6, 7). Thus, the role of mentor became one of great responsibility – bestowed on the mentee as a sort of ‘gift from the Gods’.

During the early 1900s, the forefathers of modern medicine (e.g., Osler, Cushing) modeled and advocated a close mentor–mentee relationship which, ultimately, proved hugely advantageous for both parties as well as for the burgeoning field of medicine. A mentor, according to this classic example, is a person with influence and knowledge who offers wisdom, advice, and opportunity for the mentee – a close mutually beneficial alliance requiring motivation and commitment from both parties. Traditionally, a mentor needs to possess not just superior skills and knowledge but also high moral and professional values (8).

Unfortunately, multiple studies have shown that despite the proliferation of formal and informal mentorship programs, the current situation is less than ideal. Mentorship, it appears, requires a level of time, commitment, and altruism that is not easily delivered in the current environment of decaying public trust, eroding professionalism, and financial competitiveness (9). This situation has left some mentees dissatisfied and disillusioned; for others, attuned to these new demands, mentorship is no longer ‘a gift from the Gods’ – but, rather, a question of ‘What a mentor can do for me?’.

In the 1600s, medical education was haphazard and disorganized, to say the least. Most aspiring physicians ‘apprenticed’ themselves to self-defined ‘healers’. To pass on the trade of healing, then, has been deeply engrained in medicine since the days of Galen and Hippocrates. Even today, in fields such as surgery, trainees shadow a single or a team of surgeons to glean certain parts of their training. The explicit and implicit transference of knowledge, skill, and professionalism (or lack thereof) is a key process in this apprenticeship.

How does apprenticeship compare to mentorship? A mentor imparts not only knowledge but also may be a benefactor to and/or advocate for the mentee by means of their influence in the field – offering solutions to problems or trying to obtain jobs by using their contacts, for example. As the Flexner report molded modern-day medical education, the role of apprenticeship was underplayed and a more formal, standardized approach to medical training was undertaken.

Role models are people we wish to mirror – a person who serves as an example or whose behavior is emulated by others. In sociologist Robert Merton's early work on the socialization of medical students, he hypothesized that we compare ourselves with reference groups of people who occupy the social roles to which we aspire (10). One can have numerous, different role models. Since direct contact is not necessary, the relationship need not be mutually agreed-upon. Is this sufficient for fulfilling the mentorship function? Perhaps – but as with the mentor, a mentee could unknowingly emulate values and behaviors not necessarily befitting of medical professionalism.

The balance may lie in the realization that mentorship, in its true sense, may be a fading reality incompatible with the changing educational and practice environments. On one end, the standards to which mentors are held as deserving of mentees’ emulation and respect are higher. On the other, the tangible outcomes associated with effective mentorship do not readily lend themselves to current definitions of ‘productivity’.

Training and rewarding mentors may be one answer. As in other professions, having a trained ‘coach’ may provide a more mutually fulfilling experience – someone who is able to more freely devote time to training others without competition from undue clinical/administrative duties or time constraints. Admittedly, this may be a utopian vision. Instead, we may need to train, nurture, and engender an entirely new ‘breed’ of medical educators who are able to navigate within prevailing practice demands without abandoning their roles as teachers and, more importantly, as mentors.
